# Hedgehog associated to microparticles inhibits adipocyte differentiation *via* a non-canonical pathway

**DOI:** 10.1038/srep23479

**Published:** 2016-03-24

**Authors:** Audrey Fleury, Lucile Hoch, M. Carmen Martinez, Hélène Faure, Maurizio Taddei, Elena Petricci, Fabrizio Manetti, Nicolas Girard, André Mann, Caroline Jacques, Jérôme Larghero, Martial Ruat, Ramaroson Andriantsitohaina, Soazig Le Lay

**Affiliations:** 1 INSERM U1063, Université d’Angers, IBS-IRIS Rue des Capucins, F-49100 Angers, France; 2 CNRS, UMR-9197, Neuroscience Paris-Saclay Institute, Molecules Circuits Department, 1 Avenue de la Terrasse, F-91198, Gif sur Yvette, France; 3 Dipartimento di Biotecnologie, Chimica e Farmacia, Università degli Studi di Siena, Via A. Moro 2, I-53100, Siena, Italy; 4 CNRS, UMR-7200, Laboratoire d’Innovation Thérapeutique, Université de Strasbourg, 74 Route du Rhin, BP 60024, F-67401 Illkirch, France; 5 Assistance Publique – Hôpitaux de Paris, Hôpital Saint-Louis, Unité de Thérapie Cellulaire; Inserm UMR1160 et CIC de Biothérapies; Univ Paris Diderot, Sorbonne Paris Cité, F-75475, Paris, France

## Abstract

Hedgehog (Hh) is a critical regulator of adipogenesis. Extracellular vesicles are natural Hh carriers, as illustrated by activated/apoptotic lymphocytes specifically shedding microparticles (MP) bearing the morphogen (MP^Hh+^). We show that MP^Hh+^ inhibit adipocyte differentiation and orientate mesenchymal stem cells towards a pro-osteogenic program. Despite a Smoothened (Smo)-dependency, MP^Hh+^ anti-adipogenic effects do not activate a canonical Hh signalling pathway in contrast to those elicited either by the Smo agonist SAG or recombinant Sonic Hedgehog. The Smo agonist GSA-10 recapitulates many of the hallmarks of MP^Hh+^ anti-adipogenic effects. The adipogenesis blockade induced by MP^Hh+^ and GSA-10 was abolished by the Smo antagonist LDE225. We further elucidate a Smo/Lkb1/Ampk axis as the non-canonical Hh pathway used by MP^Hh+^ and GSA-10 to inhibit adipocyte differentiation. Our results highlight for the first time the ability of Hh-enriched MP to signal *via* a non-canonical pathway opening new perspectives to modulate fat development.

The conserved family of Hedgehog (Hh) proteins, including Desert (Dhh), Indian (Ihh) and Sonic (Shh), are secreted signalling morphogens of crucial importance in the control of tissue patterning, cell growth and differentiation during development^1^. Hh is also expressed in adult tissues where it participates to physiological and pathophysiological responses^2^. Mature Hh proteins are produced as precursors that are processed through autocatalytic cleavage. This yields a secreted N-terminal peptide further modified by covalent attachments of a cholesterol molecule at its C-terminus^3^ and a palmitic acid at its N-terminus^4^. These adducts confer a high affinity for lipid-modified Hh with the plasma membrane triggering a local and high level of signalling response (for review^5^). Hh activates signalling cascade into targeting cells by binding to the multipass membrane receptor Patched (Ptch). Other Hh binding proteins, including putative co-receptors for Hh ligands, are also identified to be mandatory for correct Hh pathway activity in multiple tissues^6^. Hh binding to Ptch leads to derepression of Smoothened (Smo), a member of the class F GPCR (G-protein coupled receptors)^7^. Active Smo translocates to the tip of the primary cilium and prevents the proteolytic cleavage of the Gli-1 to Gli-3 transcription factors allowing their nuclear translocation and regulation of their target genes^8^. While this canonical Hh pathway regulates the majority of the biological effects of Hh signalling, other non-canonical pathways, independent of Gli but still relying on Smo were reported^7^,^9^,^10^. Hh ligands can then promote cytoskeleton rearrangement through a Smo-RhoA-Rac1 coupling mechanism in endothelial cells^11^,^12^. In neurons, these ligands modulate Ca^2+^ spike activity through Smo-heterotrimeric GTP-binding protein-dependent pathway^9^. They can also stimulate axon guidance through Src kinases activation *in vitro*^13^. Recently, Teperino *et al*. revealed the pivotal role of a Smo-Ampk axis (5′ adenosine monophosphate-activated protein kinase) in the regulation of cellular metabolism, notably through increased insulin-independent glucose uptake in brown fat tissue and muscle^14^. The existence of this non-canonical Hh/Ampk pathway was recently confirmed to play a critical role in polyamine metabolism in medulloblastoma cells^15^. Our previous identification of the Smo modulator propyl 4-(1-hexyl-4-hydroxy-2-oxo-1,2-dihydroquinoline-3-carboxamido) benzoate (GSA-10), which mediates its cellular effects independently of cilium Smo translocation and Gli activation^16^, highlights the fact that, depending on ligands, different Smo conformations signal through various downstream pathways.

Since membrane tethering of double-lipid modified Hh peptides precludes direct effects on distant cells, several mechanisms contribute to ligand release and subsequent transport. Although lipid-free Hh forms are detected at low levels *in vivo*^17^, the main Hh form detected in most species is cholesterol-modified lipid adduction that conditions its long-range spreading^18^. Hence, circulating lipidated forms of Hh have also been detected as soluble multimeric protein complexes^19^, or shown to associate with lipoproteins^20^,^21^ and to extracellular vesicles (EV) including exosomes and microparticles (MP)^18^,^22^,^23^,^24^,^25^,^26^. Interestingly, others and we demonstrated that MP-associated Hh ligands triggered Hh signalling in endothelial cells inducing pro-angiogenic responses^25^,^27^ and preventing apoptotic processes^28^,^29^. Moreover, intravenous injection of Hh-enriched MP (MP^Hh+^) in ischemic rodent models confirm their pro-angiogenic potential^28^,^30^ as well as their cardioprotective effects^31^ shedding light on the therapeutic potential of these MP^Hh+^ in cardiovascular diseases^32^.

Among the numerous biological effects reported for Hh morphogens^2^,^33^, Shh was identified as a critical regulator of fat development by altering white adipocyte differentiation^34^,^35^. Activation of Hh signalling in mesenchymal stem cells indeed favours osteoblastic differentiation at the expense of adipogenesis^36^. Whereas the molecular basis underlying these anti-adipogenic effects are not fully elucidated, Shh seems to act on early steps of adipocyte differentiation, through the modulation of key Gli-dependent transcriptional regulators^35^, upstream to peroxisome proliferator-activated receptor γ (PPARγ)^34^. Hh effects on adipogenesis are all based on the response obtained by recombinant protein (recShh) or the use of Smo agonists such as SAG and purmorphamine that stimulate the Hh canonical pathway^34^,^35^.

In the current report, we study the effects of MP either harbouring Hh ligands or devoid of them (MP^Hh+^ and MP^Hh−^, respectively) isolated from cultured supernatants of apoptotic/stimulated T-lymphocyte cells^23^. We show here that MP^Hh+^ specifically inhibit adipocyte differentiation. Despite similar anti-adipogenic effects, MP^Hh+^ differ from recShh or SAG by activating a different downstream signalling pathway independent of the transactivation of Gli factors. We reveal that MP^Hh+^ anti-adipogenic effects, which can be partially mimicked by the Smo agonist GSA-10, rely on the activation of a Smo/Lkb1 (Liver kinase B1)/Ampk axis. These results highlight, for the first time, the ability of Hh-enriched MP to signal *via* a non-canonical pathway to inhibit adipocyte differentiation.

## Materials and Methods

### Reagents and compounds

Unless otherwise stated, all chemicals and reagents were obtained from Sigma-Aldrich (St Louis, MO). SAG, GSA-10, GDC-0449, MRT-92 and LDE225 were synthesized as described^16^,^23^,^37^. Purmorphamine and recombinant Shh (C25II N-Term, RecShh) were purchased from Calbiochem and R&D Systems, respectively. Cyclopamine was purchased from Enzo Life Sciences and KAAD-Cyclopamine was from Santa Cruz Biotechnologies. SANT-1 was obtained from TOCRIS. SAG and cyclopamine were dissolved in ethanol, while the other compounds were dissolved in DMSO at a concentration of 10 mM, except GSA-10, which was at 2.5 mM. Unless specified, the following concentrations of these compounds were used: SAG (200 nM), GSA-10 (10 μM), recShh (0.5 μg/mL), KAAD-cyclopamine and cyclopamine (10 μM), SANT-1 (1 μM), LDE225 (3 μM), MRT-92 (1 μM) and GDC-0449 (3 μM).

### MP production

**Figure 1:**
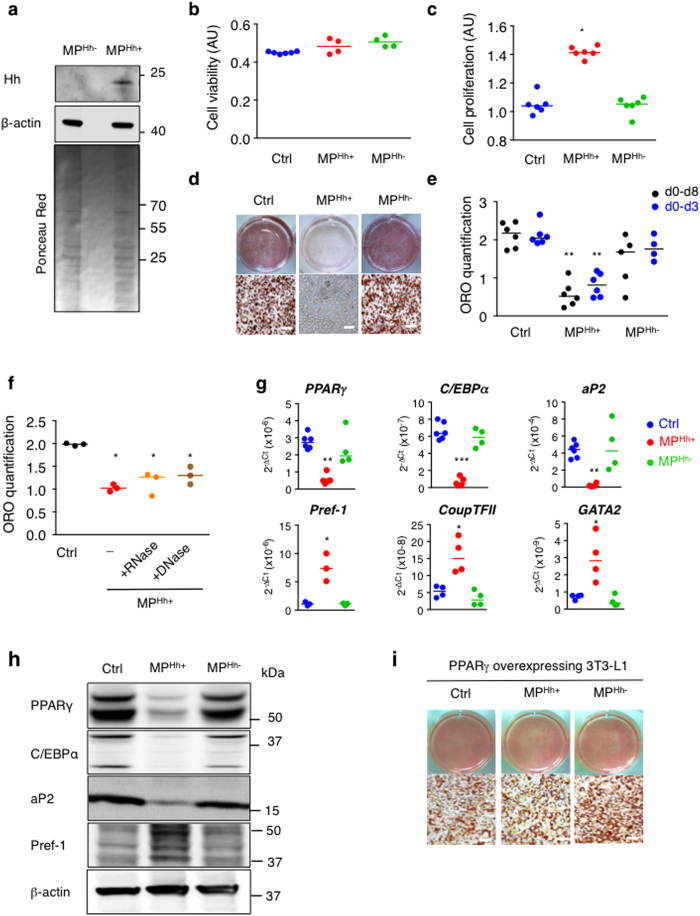
MP^Hh+^ stimulate proliferation and inhibit 3T3-L1 adipocyte differentiation. (**a**) Immunoblot showing specific association of Hh with MP isolated from supernatants of PMA/PHA/ActD-stimulated CEM-T lymphocytes (MP^Hh+^) compared to ActD-stimulated CEM-T cells (MP^Hh−^). β-actin, known to associate with MP, is used as a loading control. Ponceau red staining of the membrane shows equal protein loading for the two MP preparations. (**b**) MP^Hh+^ or MP^Hh−^ do not alter cell viability. Cell viability, estimated using the MTT test, was measured after incubation of 10 μg/mL of each MP subtype for 48 h on 3T3-L1 preadipocytes. (**c**) MP^Hh+^ significantly increase cell proliferation. 3T3-L1 DAPI-stained nuclei were counted after incubation of 10 μg/mL of each MP subtype for 72 h on 3T3-L1 preadipocytes. (**d**) MP^Hh+^ inhibit adipocyte differentiation. 3T3-L1 cells were exposed to 10 μg/mL of each MP subtype during the all course of differentiation. While the control and MP^Hh−^ -treated 3T3-L1 fully differentiate, MP^Hh+^-exposed cells show hardly no differentiated cells as indicated by the absence of ORO-stained cells. (**e**) Single exposure to MP^Hh+^ is sufficient to inhibit adipogenesis. 3T3-L1 cells were exposed to 10 μg/mL MP^Hh+^ once (on day 0 for 72 h, d0–d3) or during the all course of differentiation (d0–d8). ORO quantification (on day 8) reveals significant adipogenesis inhibition (p < 0.05) following MP^Hh+^ treatment, independently of the duration of MP^Hh+^ exposure to 3T3-L1. (**f**) Nuclease-pretreatment of MP^Hh+^ does not alter their anti-adipogenic effects. 3T3-L1 were exposed to 10 μg/mL MP^Hh+^ pre-treated or not with nucleases (RNase or DNase) for 72 h. ORO quantification was performed and compared to 3T3-L1 cells not treated with MP (Ctrl). (**g,h**) MP^Hh+^ treatment of 3T3-L1 preadipocytes significantly decreases key adipogenic factors and increases anti-adipogenic markers. mRNA expression (**g**) and protein expression (**h**) of key factors of adipocyte differentiation were evaluated on day 6 in the absence or presence of 10 μg/mL of indicated MP. Different isoforms of Pref-1 are detected. Unprocessed immunoblots, from which the presented images were cropped, are presented Fig. S6. (i) Stable retroviral expression of PPARγ reverses MP^Hh+^-dependent inhibition of 3T3-L1 adipogenesis. Representative images of ORO-stained cells are shown, *n* = 2 independent experiments. Scale bar: 50 μm.

MP were produced from CEM T cells as previously described^23^. Briefly, one million cells/ml were either treated with actinomycin D (ActD, 1 μg/mL) for 24 h or treated with phytohemagglutinin (PHA, 10 μg/mL) for 72 h. The cells were then treated with phorbol-12-myristate-13 acetate (PMA, 40 ng/mL) and ActD (1 μg/mL) for 24 h. Under these conditions, 0.12 ± 0.02 μg of MP^Hh−^ and 0.25 ± 0.07 μg of MP^Hh+^ were secreted by 10^6^ CEM T cells following respectively stimulation by either Act D alone or ActD/PMA/PHA combined treatment. These different stimulation protocols led to the production of MP with or without Hh (Fig. 1a). Supernatants were obtained by centrifugation at 750 *g* for 20 min and then at 1,500 *g* for 5 min to remove cells and large debris, respectively. MP pellets were recovered from supernatants by two repetitive centrifugations (45 min at 13,000 *g*), washed in 0.9% NaCl and resuspended in 200 μl of 0.9% NaCl solution. Exosome pellets were further isolated from MP-depleted supernatants by a 100,000 *g* centrifugation step for 1 h at 4 °C, followed by two wash steps with NaCl (100,000 *g*, 1 h 4 °C), and resuspended in 0.9% NaCl. For experiments using 5E1 blocking antibody, MP^Hh+^ (10 μg/mL) was preincubated with 10 μg/mL 5E1 blocking antibody (Developmental Studies Hybridoma Bank, Iowa City, IA) for 30 min at 37 °C in DMEM 10% FBS medium, before incubating with the cells. To remove nucleic acids, MP were treated with DNaseI or RNaseA as previously described^29^. The efficiency of nuclease treatment was evaluated by MP-RNA/DNA analyses using the Agilent 2100 bioanalyzer (Agilent Tech. Inc., Santa Clara, CA) to confirm the absence of residual nucleic acids.

### Cell culture

3T3-L1 cells were maintained in high-glucose Dulbecco’s modified Eagle’s medium (DMEM) with 10% donor calf serum at 37 °C and 10% CO_2_. Once seeded in 6-well-plates, they were induced to differentiate two-days post-confluence in classical medium in the presence of 10% foetal bovine serum (FBS), 250 μM 3-Isobutyl-1-methylxanthine (IBMX), 1.25 μM dexamethasone (Dex) and 250 nM insulin for three days, and then cultured with 100 nM insulin alone until complete adipocyte differentiation (day 6–8). In minimal induction medium conditions, two-day old post-confluent cells were treated with 5 μM rosiglitazone and 870 nM insulin for three days followed by incubation with 100 nM insulin alone until complete differentiation. One million 3T3-L1 cells were treated with MP or compounds as indicated in the figure legends in 1.5 mL induction medium. The amount of MP corresponding to 10 μg/mL used for 3T3-L1 treatment resulted to MP production from either 132 ± 20 millions ActD-stimulated CEM T cells or from 66 ± 18 millions PHA/PMA/ActD-stimulated CEM T cells, respectively. Therefore, the ratio of MP-secreting T lymphocytes to 3T3-L1 recipient cells has been estimated to 132:1 (in the case of MP^Hh−^) and 66:1 (in the case of MP^Hh+^). Such ratio is particularly relevant in the context of obesity, since cells from stroma vascular fraction (SVF) (usually in a 1:1 ratio with adipocytes in lean conditions^38^) significantly increased, notably due to the recruitment of immune cells. Hence, in obese adipose tissue, T lymphocytes population accounts for 20% of SVF^39^.

Human mesenchymal stem cells (hMSCs) were isolated from washed filters used during bone marrow graft processing. Experimental procedures using human bone marrow cells were approved by Saint Louis Hospital Ethical Committees for human research (Paris, France) and all experiments were performed with relevant guidelines and regulations. hMSCs were obtained and cultured as previously described^40^. Briefly, bone marrow cells from healthy donors obtained after Ficoll purification (Invitrogen Corporation, San Diego, CA, USA) were cultured in α-minimum essential medium, supplemented with 10% FBS (Gibco, Invitrogen Corporation), 1% Penicillin/Streptomycin (Gibco, Invitrogen Corporation) and 1 ng/mL basic fibroblast growth factor (PeproTech, Neuilly sur Seine, France). At confluence, cells were rinsed with low-glucose DMEM and placed in adipogenic medium composed of DMEM 20% FBS with 1 μM dexamethasone, 500 μM IBMX, 870 nM insulin and 60 μM indomethacin. Based on the maximal effects obtained with this concentration on the adipocyte differentiation of 3T3-L1 cells, 10 μg/mL of CEM T-derived MP were incubated with hMSCs during the entire differentiation process. After two weeks, the protein expression of key transcription factors for adipogenesis was evaluated by Western blot analysis.

The human lymphoid CEM T cell line (American Type Culture Collection, Manassas, VA) was used for MP production. Cells were seeded at 10^6^ cells/mL and cultured in serum-free X-VIVO 15 medium (Lonza, Walkersville, MD).

### Small-hairpin RNA (ShRNA)

Ready-to-use lentiviral particles (MISSION^®^ from Sigma, pLKO.1 plasmid backbone) expressing shRNA targeting Smo (TRCN0000026245), Ampkα1 (TRCN0000024003), Lkb1 (TRCN0000024146) or Camkk2 (TRCN0000276649) were used at a multiplicity of infection of 20 in complex with 8 μg/mL polybrene to transduce the growth of 3T3-L1 pre-adipocytes. Non-target lentiviral particles expressing a scramble (Scr) shRNA (MISSION^®^ pLKO.1-puro Non-Target shRNA Control Transduction Particles, #SHC016V) were used to generate 3T3-L1 control cell lines. Puromycin (6 μg/mL) was used to select stable 3T3-L1 fibroblast clones, which were further allowed to differentiate into adipocytes, as described above. SybrGreen real-time qPCR using primers listed in Table S1 was used to validate the knockdown (Kd) efficiencies in 3 independent experiments. The remaining expression levels of targeted genes (compared to gene expression in Scr 3T3-L1 used as control cells) were as follows: 20.2 ± 7.1% for Lkb1 Kd; 23 ± 5.5% for Camkk2 Kd and 18.6 ± 3% for Smo Kd.

pBabe retroviruses were produced as previously described^41^ and stably-infected 3T3-L1 selected as detailed above. 3T3-L1 overexpressing PPARγ were cultured to confluence in DMEM 10% FBS and insulin (870 nM). At confluence, cells were induced to differentiation with dexamethasone (625 nM) and a PPARγ activator (1.2 μM rosiglitazone) in the presence or absence of 10 μg/mL MP^Hh+^.

### Oil Red staining

Lipid accumulation was quantified by Oil Red O (ORO) staining. Briefly, 3T3-L1 cells (day 6–8 of differentiation) were fixed with 4% paraformaldehyde (PFA, Electron Microscopy Sciences, Hatfield, PA) and incubated with freshly prepared 0.2 μm-filtered ORO solution (one part of water mixed with three parts of 1 g/100 mL ORO solution in isopropanol) for 30 min. Extensive washing with PBS removed the excess stain. ORO was extracted from the stained cells using 100% isopropanol. The staining was quantified by measuring the optical density at an absorbance of 510 nm.

### Cell viability and proliferation

The MTT (3-{4,5-dimethylthiazol-2yl-2,5-diphenyltetrazolium bromide) assay was used to evaluate the toxicity of CEM T-derived MP on 3T3-L1 cells. After 6 hours incubation with MTT (25 μg/mL), at 37 °C in a 10% CO_2_ atmosphere, an MTT detergent solution was added for 12 h at 37 °C. The optical density of the solution was measured at 570 nm to evaluate cell viability. Cell proliferation following 72 h MP treatment was estimated by counting 4′-6 diamidino-2-phenyl indole-2HCl (DAPI)-stained nuclei of the fixed 3T3-L1 cells per microscopic field. Five independent microscopic fields were quantified.

### MP internalization

MP were stained with PKH26 dye (Sigma Aldrich) following the manufacturer’s protocol. Briefly, MP were labelled with 2 μM PKH26 dye in 0.9% NaCl solution for 2 min at room temperature. An equal volume of FBS was added to stop the staining reaction. The MP pellet was recovered by centrifuging at 14,000 *g* for 45 min, washing in 0.9% NaCl, and was finally resuspended in 0.9% NaCl. This labelling procedure was efficient as confirmed by flow cytometry (93.5 ± 3.4% of MP population PKH26^+^, *n* = 4 independent labelling experiments).

Post staining, 10 μg/mL PKH26-labeled MP^Hh+^ were incubated with 3T3-L1 preadipocytes for the indicated times either at 37 °C or 4 °C. At the end of the incubation time, MP internalization in 3T3-L1 was evaluated either by flow cytometry on trypsinized 3T3-L1 cells resuspended in 250 μl PBS solution (500 MPL system, Beckman Coulter, Villepinte, France), or by confocal microscopy of PFA fixed and DAPI counterstained cells.

### Alkaline Phosphatase (ALP) assay

hMSC cells were incubated in the presence of MP^Hh+^ in adipogenic medium for 2 weeks. Naphtol-AS-BI phosphate (Sigma-Aldrich, 1.25 mM) was dissolved in 0.2 M Tris-HCl buffer, pH 8.5 and mixed with 2.5 mM Fast blue BB (Sigma-Aldrich). 4% PFA-fixed-hMSC cells were incubated with this mixture for 2 h at 37 °C. Cells were washed three times with PBS and incubated with 0.1 M NaF (Sigma-Aldrich) for 30 min. Cells were then rinsed twice with PBS followed by two rapid washes with distilled water. The percentage of cells positive for ALP (blue) staining was quantified in four independent microscopic fields.

### Immunoblotting

Protein lysates either from cells, MP or exosomes were prepared using the previously described lysis buffer^41^. The protein concentration was determined using a Quick-Start Bradford Bio-Rad kit and samples were subjected to SDS-PAGE on 4–12% Bis-acrylamide resolving gels (Novex^®^ NuPAGE^®^ precasted gels, Life Technologies, Saint Aubin, France). 15 μg of cell lysates or 20 μg of MP or exosomal preparations were diluted with 6X Laemmli Buffer before loading on the gel. The separated proteins were transferred onto nitrocellulose) llulose membranes (GE Healthcare, Pittsburg, PA) and blotted according to standard procedures. Protein signals were visualized using enhanced chemiluminescence (Immunocruz, Santa Cruz Biotechnology, Dallas, Texas) with a Chemi-Smart 5000 imager system (Vilber-Lourmat, Marne-la-Vallée, France).

The following primary antibodies diluted in TBS/0.1% Tween, 5% BSA were used: β-actin (1:1000; Sigma; #5316), anti-total Ampkα (1:1000; CST; #2532), anti-phospho Ampkα (Thr172) (1:1000; CST; #2535), anti-C/EBPα (1:1000; SC biotech; sc-61), anti-aP2 (1:1000; CST; #2120), anti-CD63 (1:1000; SC; #SC-15363), anti-PPARγ (1:1000; CST; #2435), anti-Pref-1 (1:1000; CST; #2069), anti-Hh (1:1000 rabbit antiserum)^42^, anti-phospho Ser^473^ Akt (1:1000; CST, #3827), anti-total Akt (1:1000; CST, #3198), anti-LC3 (1:1000; CST, #4108). For detection of Ampk and LC3 signals, 3T3-L1 cells were starved overnight in high glucose DMEM supplemented with 1% fatty acids free-BSA. The medium was then replaced with fresh starvation medium supplemented with 10 μg/mL MP^Hh+^ or the indicated compounds. Immunoblots were quantified using ImageJ software.

### Gli-dependent luciferase activity

Gli-dependent reporter assay was performed as previously described^37^ in Shh-light2 cells stably expressing the Gli-Firefly and renilla reporter^43^. Cells were incubated with the drug to be tested and the luciferase activities were measured 40 h later. The data obtained were the readings of the firefly luciferase activity compared to the Renilla control activity.

### RNA extraction and real-time qPCR

3T3-L1 cells treated with either MP or different compounds during the induction cocktail phase were processed at day 6 for RNA extraction using the RNeasy mini kit (Qiagen, Courtaboeuf, France). Total RNA (500 ng) was reverse transcribed using random hexamers and oligodT using PrimeScript^TM^ RT reagent Kit (Takara/Clontech, Mountain View, CA). SybrGreen primers for the tested genes are listed in Supplemental Table 1 (Table S1). Real-time qPCR was conducted, using 40 ng cDNA and both the forward and the reverse oligonucleotides, in a 20 μl final volume using the iQSybrGreen Supermix (BioRad). Amplification curves were monitored and assessed in a CFX96 thermocycler (Biorad). All values were normalized to 36B4 expression.

### Confocal microscopy

3T3-L1 preadipocytes were seeded on coverslips and incubated with MP or chemical compounds for the indicated time and concentrations. Cells were then fixed with 4% PFA in PBS for 20 min, washed, permeabilized with 0.05% Triton X-100 PBS solution for 5 min, and washed in a blocking gelatin-PBS (0.2%) solution. For cilium staining, anti-acetylated tubulin (1:1000; Sigma, #T7451) diluted in the blocking solution supplemented with 1% BSA was incubated on cells for 2 h at room temperature. Cy3-coupled secondary antibody was added (1:1000) in fresh blocking solution for 90 min at room temperature following gelatin-PBS washes. Cells were washed again in gelatin-PBS solution, stained with DAPI, mounted in Mowiol and visualized by confocal laser fluorescence (Zeiss LSM 710). Acetylated-tubulin (red) positive cells were analysed in five representative microscopic fields at the 20X objective and compared to total cells (DAPI staining). The percentage of ciliated cells was evaluated during the course of 3T3-L1 differentiation or following MP or compound treatments.

### Statistical analysis

All experimental data are presented as scatterplots plus median to provide full information about the variability of data sets^44^. Statistical analysis of the results was performed by using non-parametric tests such as Mann-Whitney or Wilcoxon signed-rank test, respectively for comparison of non-paired or paired samples. The differences were considered significant when *p* value <0.05 and stated as follow: *for *p *< 0.05, **p < 0.01 and ***p < 0.001.

## Results

### MP carrying Hh (MP^Hh+^) specifically inhibit 3T3-L1 adipocyte differentiation

MP harbouring membrane-derived Hh morphogen were isolated from supernatants of CEM T lymphocytic cells following PHA/PMA/ActD treatment, as previously described^23^. Western blot analysis revealed that these activated/apoptotic lymphocytes specifically shed MP bearing Hh (MP^Hh+^), whereas apoptotic-T lymphocytic cells secreted MP that do not carry this morphogen (MP^Hh−^) when stimulated with ActD alone (Fig. 1a).

The concentration-response to CEM T-derived MP on adipocyte differentiation was conducted in 3T3-L1 cells differentiated according to a classical induction protocol (see methods) upon addition of these MP at each medium change (every two days). We first checked that MP^Hh+^ treatment at the highest concentration tested (10 μg/mL) did not affect cell viability as investigated by the MTT test (Fig. 1b). In contrast, it significantly increased the proliferation of 3T3-L1 preadipocytes (Fig. 1c). Exposure of 3T3-L1 preadipocytes to 10 μg/mL MP^Hh+^ specifically inhibited adipocyte conversion as illustrated by decreased ORO staining (Fig. 1d). The concentration of 10 μg/mL MP^Hh+^ was the most effective since treatment of 3T3-L1 with lower doses of MP^Hh+^ displayed less inhibitory potential (Fig. S1a). However, 10 μg/mL MP^Hh−^ did not affect 3T3-L1 adipogenesis at any of the tested concentrations (Fig. 1d, Fig. S1a) thereby excluding an inhibitory effect of MP treatment. Exosomes isolated from MP-depleted supernatants of ActD or PHA/PMA/ActD-treated CEM T lymphocytes were both devoid of Hh (Fig. S1b) and did not inhibit adipocyte differentiation (see Fig. S2c). We also established that a single treatment of 3T3-L1 with 10 μg/mL MP^Hh+^ during the induction phase of adipocyte differentiation was sufficient to obtain the maximal inhibitory effect on lipid accumulation (Fig. 1e), suggesting that MP^Hh+^ act at the early stages of adipogenesis. Nuclease-pretreatment of MP^Hh+^ did not prevent their inhibitory effects demonstrating that the anti-adipogenic effect triggered by MP^Hh+^ were independent of nucleic acids transfer from MP (Fig. 1f).

Adipogenesis inhibition following MP^Hh+^ exposure was associated with a marked reduction of mRNA expression of key adipogenic transcription factors, PPARγ and C/EBPα, and the terminally differentiated marker aP2, whereas C/EBPβ mRNA expression remained unmodified (Fig. 1g). Conversely, mRNA expression levels of anti-adipogenic markers like Pref-1, Coup-TFII and GATA-2 were specifically induced by MP^Hh+^ treatment (Fig. 1g). In line with this, PPARγ, C/EBPα and aP2 proteins levels were specifically decreased while Pref-1 protein levels increased following MP^Hh+^. Incubation with MP^Hh−^ in contrast, did not modify the protein expression of these key adipogenic factors (Fig. 1h) and did not change the mRNA expression of both adipogenic and non-adipogenic markers (Fig. 1g). Finally, MP^Hh+^ were ineffective in blocking adipogenesis in 3T3-L1 stably overexpressing PPARγ, suggesting that MP^Hh+^ trigger anti-adipogenic events upstream of PPARγ (Fig. 1i). Hence, Hh-association to MP triggers specific inhibition of 3T3-L1 adipocyte differentiation by acting at early stages, upstream of PPARγ activation.

### MP^Hh+^ induce a pro-osteogenic program in human mesenchymal stem cells

**Figure 2:**
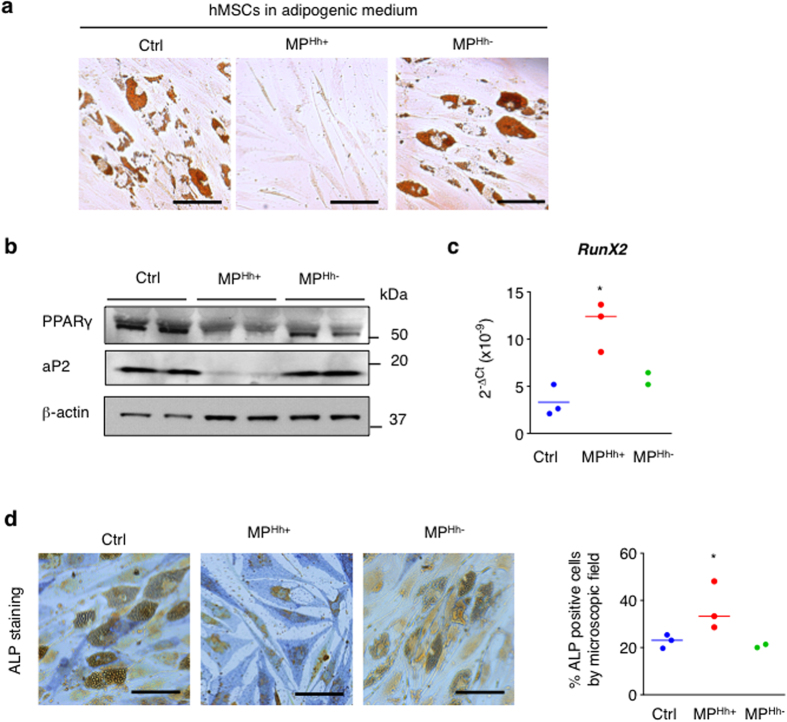
MP^Hh+^ induce a pro-osteogenic program in human MSCs. (**a**) MP^Hh+^ inhibit adipocyte differentiation of human mesenchymal stem cells (hMSCs). hMSCs were placed in adipogenic conditions and incubated with 10 μg/mL of MP^Hh+^ or MP^Hh−^. Represented images of ORO-stained cells are shown, *n* = 2 independent experiments (scale bar: 50 μm). (**b**) MP^Hh+^ alter impair protein expression of key adipocyte factors of hMSCs-differentiated adipocytes. Unprocessed immunoblots, from which the presented images were cropped, are presented Fig. S7. (**c,d**) MP^Hh+^ induce the expression of osteogenic markers in hMSCs. hMSCs were incubated with 10 μg/mL MP^Hh+^ in adipogenic conditions. mRNA expression of transcription factor Runx2 was assessed by real-time qPCR (**c**) and alkaline phosphatase activity (ALP) was evaluated following ALP staining assay (**d**). Representative images of ALP-stained cells are shown (scale bar: 50 μm).

Since CEM T cells are of human origin, we wanted to exclude the possibility that the inhibitory phenotype observed following treatment of murine 3T3-L1 with MP^Hh+^ was the result of inter-species reactivity responses. We therefore investigated the effect of MP^Hh+^ on a human model of adipocyte differentiation, namely human mesenchymal stem cells (hMSCs) cultured in adipogenic conditions. As shown in Fig. 2a, MP^Hh+^ also interfered with hMSCs-adipocyte differentiation compared to vehicle or MP^Hh−^ treatment. Similar to 3T3-L1, decreased PPARγ and aP2 protein expression levels evidenced adipogenesis blockade in hMSCs (Fig. 2b). Since Hh influences the balance between adipogenesis and osteogenesis, we investigated whether MP^Hh+^ could promote an osteogenic program in these hMSCs. Despite the adipogenic culture conditions, MP^Hh+^ treatment induced a significant increase in Runx2 mRNA expression, an early transcription factor in osteogenesis, whereas MP^Hh−^ treatment had no effects (Fig. 2c). In line with this, MP^Hh+^ incubation induced an elongated osteoblast-shape phenotype (see Fig. 2a) that was associated with an increase in alkaline phosphatase (ALP) activity (Fig. 2d). MP^Hh−^ -treated hMSCs still keep an adipogenic cell profile as illustrated by the maintenance of many lipid droplets in hMSCs-differentiated cells as in control cells (Fig. 2d). Altogether, these results confirm that Hh influences the balance between adipogenesis and osteogenesis even in its MP-associated form.

### Adipogenesis inhibition mediated by MP^Hh+^ is independent of Gli transcription factors

**Figure 3:**
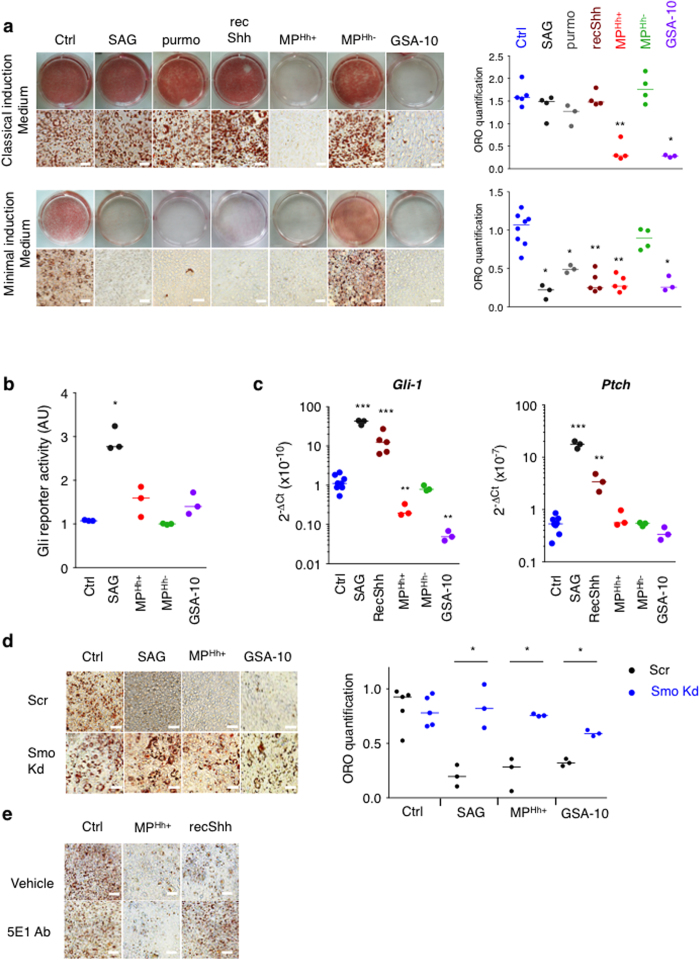
Adipogenesis inhibition induced by MP^Hh+^ and by the Smo agonist GSA-10 is independent of the Hh canonical pathway. (**a**) Anti-adipogenic effects of MP^Hh+^ or GSA-10 are not dependent on the presence of IBMX or dexamethasone in the induction medium, contrary to that of SAG, purmorphamine or recShh. ORO staining of 3T3-L1 cells induced with classical (IBMX/Dex/Ins) or minimal (Rosi/Ins) differentiation cocktails in the absence (Ctrl) or presence of SAG (200 nM), purmorphamine (purmo, 10 μM) and recShh (0.5 μg/mL), 10 μg/mL MP^Hh+^ or MP^Hh−^ and GSA-10 (10 μM). Representative images of ORO-stained cells are shown (scale bar: 50 μm). (**b**) Differential effects of MP and Smo agonists SAG and GSA-10 on Gli-dependent luciferase reporter activity in Shh-light2 cells was measured in the presence of SAG (1 μM), GSA-10 (3 μM), MP^Hh+^ (10 μg/mL) and MP^Hh−^ (10 μg/mL). SAG but not MP^Hh+^, MP^Hh−^ nor GSA-10 induces Gli-luciferase activity, expressed as fold change (FC) compared to cells treated with vehicle alone.(**c**) mRNA expression levels of Hh pathway genes tested by real-time qPCR in response to MP^Hh+^, MP^Hh−^ and Smo agonists SAG and GSA-10 in minimal induction medium. Compound concentrations were similar to those in (**a**). SAG and recShh induced a strong induction of *Gli1* and *Ptch* mRNA expression (10–40 fold increase relative to control cells, log scale) whereas MP^Hh+^ or GSA-10 display no inductive effect on Gli-transcriptional activity. (**d**) Smo knockdown abrogates inhibition of differentiation induced by SAG, MP^Hh+^ and GSA-10. 3T3-L1 cells stably expressing scrambled shRNA (Scr) or Smo shRNA (Smo Kd) were treated in minimal induction medium with SAG (200 nM), MP^Hh+^ (10 μg/mL) or GSA-10 (10 μM) during the induction period. ORO staining and quantification show reversion of adipocyte differentiation blockade induced by all Hh signalling inducers in Smo Kd 3T3-L1 compared to Scr 3T3-L1 cells. (**e**) 5E1 Ab blocks anti-adipogenic effects of recShh, but not those induced by MP^Hh+^. 3T3-L1 cells were incubated with recShh (0.5 μg/mL) or MP^Hh+^ (10 μg/mL), both reagents preincubated with or without 5E1 Ab (10 μg/mL) for 30 min. Representative images are shown, *n* = 2 independent experiments (scale bar: 100 μm).

Our data show that MP^Hh+^ inhibit adipogenesis in classical induction medium containing key inducers of differentiation, namely phosphodiesterase inhibitor isobutylmethylxanthine (IBMX) and dexamethasone (Dex). However, previous studies established that IBMX and Dex interfere with Hh signalling abolishing the anti-adipogenic effect of the canonical Smo agonist SAG or the recombinant Shh protein (recShh)^34^,^35^ notably by decreasing Gli1 expression, a key transcriptional relay for canonical Hh signalling activity^35^,^45^. We then compared the effects of Hh pathway modulators (recShh, SAG, purmorphamine, GSA-10) to that of MP^Hh+^ on 3T3-L1 adipogenesis in classical induction medium or minimal induction medium (only containing Rosiglitazone and Insulin). Treatment of 3T3-L1 with 200 nM SAG, 10 μM purmorphamine or 0.5 μg/mL recShh had no effect on adipocyte differentiation in the classical induction medium (Fig. 3a). Inhibitory properties of these Hh canonical inducers were only observed in the minimal induction medium (Fig. 3a), 10 nM SAG is sufficient to inhibit adipocyte differentiation in these culture conditions (Fig. S2a). Addition of IBMX or Dex to minimal induction medium was sufficient to abolish SAG and recShh inhibitory effects on adipogenesis while MP^Hh+^ retained their complete inhibitory activity (Fig. S2b). MP^Hh−^, as well as exosomes isolated either from ActD or PMA/PHA-ActD-treated CEMs T lymphocytes, all devoid of Hh (see Fig. S1b), had no inhibitory effects whatever the induction medium used (Fig. 3a and Fig. S2c). Conversely, GSA-10 treatment of 3T3-L1 inhibited differentiation in classical as well as minimal induction medium similar to MP^Hh+^ treatment (Fig. 3a), with maximal effect reached at 10 μM (Fig. S2a). Moreover, MP^Hh+^ and GSA-10 were able to overcome the effect of the presence of IBMX/Dex (Fig. S2b). Thus, the conserved inhibitory potential of MP^Hh+^ in classical induction medium could be mimicked by GSA-10, but not by the Smo agonist SAG or the recombinant Hh protein.

We found that MP^Hh+^, like GSA-10, also failed to enhance Gli-reporter activity in Shh-Light2 cells in comparison to SAG (Fig. 3b). Moreover, real-time qPCR analysis of Gli target genes (including *Gli1* and *Ptch*) revealed a strong induction in minimal induction medium following SAG and recShh stimulation (10–40 fold increase) (Fig. 3c). In contrast, neither MP^Hh+^ nor MP^Hh−^ increase the transcription of these two genes, similar to GSA-10. Instead GSA-10 decreased *Gli1* mRNA expression in accordance to what we previously described in C3H10T1/2 cells^16^ (Fig. 3c). Canonical Hh signalling, initiated by Hh binding to Ptch, precedes Smo accumulation in the primary cilium and the activation of downstream Gli transcription factors^33^. We first studied whether treatment with MP^Hh+^ or Smo agonists alter the dynamics of the cilium present in 3T3-L1 cells by visualizing the acetylated tubulin-positive signal. As previously described^46^, we confirmed the transient presence of a cilium in preconfluent preadipocytes (day 2) and during the induction phase of differentiation (day 0/day 1/day 3) followed by its disappearance in fully differentiated adipocytes (day 6) (Fig. S3a). Thus, neither MP^Hh+^, SAG, nor GSA-10 treatment affected the presence of this cilium in preadipocytes (Fig. S3b) excluding the possibility that the differential responses of MP^Hh+^ and GSA-10, by comparison to SAG, may rely on an alteration of this sensory organelle.

We next questioned about Smo requirement in MP^Hh+^ mediating effects. Knockdown of Smo reversed the anti-adipogenic effects of SAG but also the effects of MP^Hh+^ and GSA-10 on 3T3-L1 cultured in minimal induction medium (Fig. 3d). These data clearly establish that MP^Hh+^, like SAG or GSA-10, stimulate Smo activity to exert their inhibitory properties. However, MP^Hh+^, like GSA-10, mediate their anti-adipogenic effects independently of the increased transcription of *Gli1*, suggesting the activation of a non-canonical Hh pathway.

The neutralizing monoclonal antibody 5E1 (5E1 Ab) binds Hh at its pseudo-active site thereby preventing Hh binding to Ptch and antagonizing the ligand-dependent pathway activation^47^. To investigate whether such binding between MP^Hh+^ and Ptch is necessary to mediate anti-adipogenic effects, we treated 3T3-L1 cells with 10 μg/mL MP^Hh+^ or recShh preincubated in the presence or absence of 5E1 Ab. Preincubation of recShh with 5E1 Ab completely abolished the anti-adipogenic potential of the recombinant protein illustrating the requirement of recShh binding to Ptch for mediating adipogenesis blockade (Fig. 3e). Surprisingly, preincubation of MP^Hh+^ with Hh neutralizing Ab did not alter their anti-adipogenic potential (Fig. 3e). Using PKH26 labelled-MP^Hh+^, we further demonstrated the ability of 3T3-L1 to rapidly internalize MP^Hh+^, with more than half of MP^Hh+^ already detected in intracellular compartments after 6 hours of incubation (Fig. S4a). Labelled-MP^Hh+^ uptake, visualized by confocal microscopy over time, was prevented by incubating the cells at 4 °C suggesting an energy-dependent process rather than a passive membrane passage (Fig. S4b). Moreover, excess of unlabelled MP^Hh+^ prevented the internalization of PKH26 labelled-MP^Hh+^ in 3T3-L1 cells supporting the specificity of this process (Fig. S4c). Finally, we observed no effect of 5E1Ab preincubation on PKH26-labeled MP^Hh+^ uptake investigated at 24 h in 3T3-L1 cells (Fig. S4d) suggesting that the MP internalization process is independent of a direct binding of Patched to Hh carried by MP. Altogether, these results demonstrate that MP^Hh+^ neither bind to Ptch *via* the Hh pseudo-active site to inhibit 3T3-L1 adipocyte differentiation nor do they use this receptor for internalization by 3T3-L1 cells.

### MP^Hh+^ and RecShh exhibit distinct pharmacology of Smo

**Figure 4:**
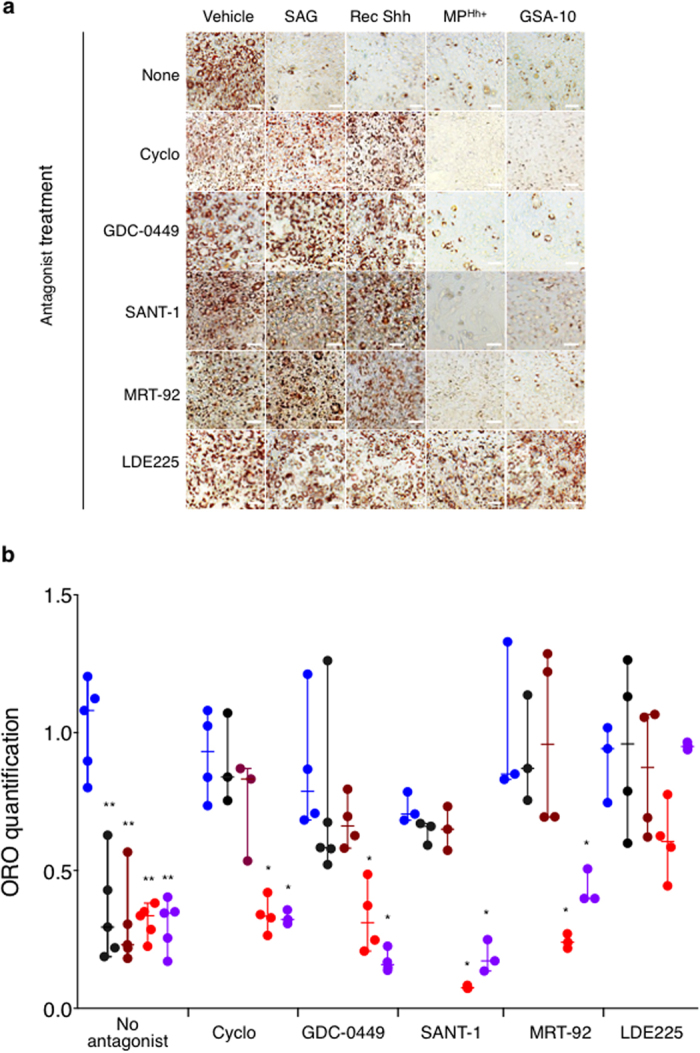
LDE225 Smo antagonist reverses adipogenesis blockade in MP^Hh+^ and GSA-10-treated 3T3-L1 cells. (**a,b**) Effects of Smo antagonists (Cyclopamine, 10 μM; GDC-0449, 3 μM; SANT-1, 1 μM; MRT-92, 1 μM; LDE225, 3 μM) added to 3T3-L1 cells concomitantly incubated with SAG, recShh, MP^Hh+^ and GSA-10 (at similar concentrations to those used for Fig. 3a) in minimal induction conditions were compared to vehicle-treated 3T3-L1 (no antagonist). Representative ORO-stained images of these experiments are presented in (**a**) and ORO-quantification results (**b**) are presented as dots with median plus range from *n* = 3–5 independent experiments.

To further characterize MP^Hh+^ activation of Smo, we investigated whether effects on 3T3-L1 cell differentiation could be inhibited by Smo antagonists with different chemical structures (cyclopamine, GDC-0449, SANT-1, MRT-92, LDE225)^7^,^37^,^48^. Noteworthy, antagonists tested were described to bind to different sites of Smo: cyclopamine, GDC-049 and LDE225 mostly bind to the 3^rd^ extracellular loops (ECL) of Smo (referred to as site 1) whereas SANT-1 binds to a narrow and deep hydrophobic cavity in its 7 transmembrane (TM) domain namely site 2^7^,^37^,^49^. Of note, we recently described binding of MRT-92 to both sites^37^. None of the tested antagonists altered the basal 3T3-L1 adipocyte differentiation (Fig. 4a). Of interest, anti-adipogenic effects of SAG and recShh in minimal induction medium were completely antagonized by all Smo antagonists tested as illustrated by the reversion of lipid accumulation (Fig. 4a,b). Conversely, none of Smo site 1 and site 2 binding antagonists prevented MP^Hh+^ or GSA-10 anti-adipogenic effects. Interestingly, LDE225, in contrast to the other tested antagonists, prevented the inhibitory effect of MP^Hh+^ and GSA-10 on adipocyte differentiation (Fig. 4a,b). Results similar to those of cyclopamine were obtained with its analog KAAD-cyclopamine (10 μM, data not shown). Such differential antagonism suggest that MP-associated Hh may act through a Smo conformation analog that binds GSA-10 and not through a Smo conformation linked to the canonical pathway activated by SAG or recShh.

### Anti-adipogenic effects of MP^Hh+^ and GSA-10 are mediated by a Smo/Lkb1/Ampk pathway

We next investigated which signalling pathways downstream of Smo were involved in MP^Hh+^ adipogenesis inhibitory effects. We therefore searched for mechanisms described for modulating adipogenesis with a link to Hh signalling.

First, we studied autophagy in response to MP^Hh+^ since this process was shown to be required for adipocyte conversion^50^,^51^, and is a crucial node for Hh signalling^52^,^53^. We analysed the flux through the autophagy pathway using bafilomycin-A1 (BafA1), a blocker of lysosomal degradation, by measuring changes in the levels of lipid-modified LC3 form, namely LC3-II. It is an autophagosome component degraded in lysosomes (Fig. S5a). Since LC3-II/LC3-I ratios were not significantly different after 10 μg/mL MP^Hh+^ exposure in the presence or absence of BafA1 (Fig. S5b), we concluded that MP^Hh+^ did not modify the basal autophagic process or the autophagic flux in 3T3-L1 cells.

**Figure 5:**
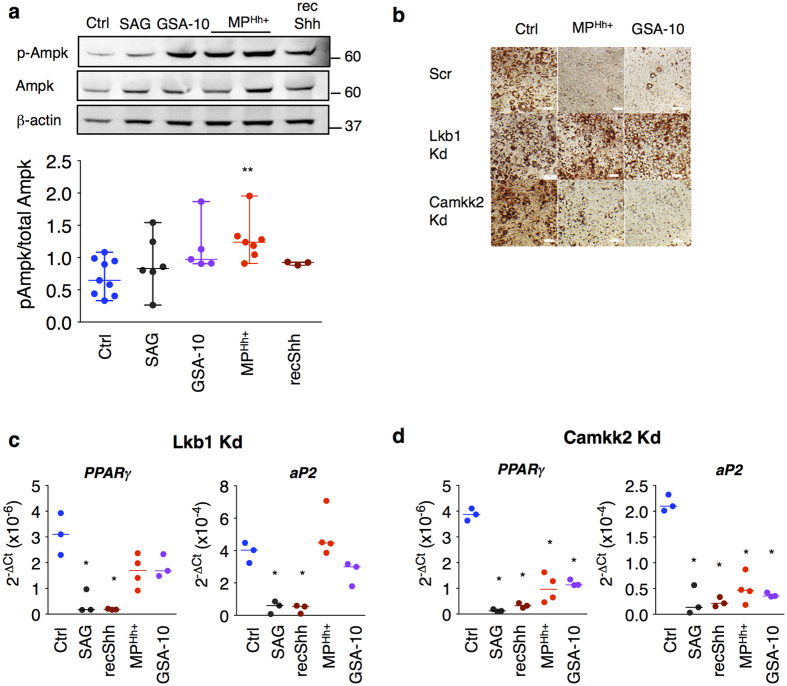
MP^Hh+^ and GSA-10 induce inhibition of adipocyte differentiation through activation of Ampk signalling pathway. (**a**) Western-blot showing increased phosphorylation of Ampk in MP^Hh+^ (10 μg/mL) and GSA-10-treated 3T3-L1 preadipocyte cells for 24 h. Following compound concentrations were used: SAG (200 nM), GSA-10 (10 μM), MP^Hh+^ (10 μg/mL) and recShh (0.5 μg/mL). β-actin immunoblot serves as a loading control. Unprocessed immunoblots, from which the presented images were cropped are presented Fig. S8. Immunoblots quantification was performed from at least *n* = 3 independent experiments. P-Ampk/total Ampk ratio are presented as experimental data dots with median plus range from *n* = 3–7 independent experiments. (**b**) Lkb1 silencing prevents from MP^Hh+^ and GSA-10-induced anti-adipogenic effects as illustrated by restored lipid accumulation. 3T3-L1 stably expressing scrambled shRNA (Scr), Camkk2 shRNA (Camkk2 Kd) or Lkb1 shRNA (Lkb1 Kd) and the control cell line were treated with 10 μg/mL MP^Hh+^ or 10 μM GSA-10 during the induction cocktail in minimal induction medium. Representative ORO-stained images of 3T3-L1-treated cells (day 6 of differentiation) are presented, *n* = 2 independent experiments. (**c**,**d**) Lkb1 knockdown abrogates inhibition of differentiation induced by MP^Hh+^ and GSA-10, whereas Camkk2 knockdown has no effects. Camkk2 Kd or Lkb1 shRNA 3T3-L1 cells were treated or not treated (Ctrl) with 10 μg/mL MP^Hh+^ or 10 μM GSA-10 during the induction period. PPARγ and aP2 mRNA expression was analysed by real-time qPCR analysis. Lkb1 Kd abrogates inhibition of differentiation induced by MP^Hh+^ or GSA-10 as illustrated by reversed mRNA expression of PPARγ and aP2. (**c**) whereas inhibition of the mRNA expression of these two genes is still maintained in Camkk2 Kd cells. (**d**). Conversely, neither Lkb1 nor Camkk2 silencing reverse anti-adipogenic effects of SAG (200 nM) or recShh (0.5 μg/μl) illustrated by conserved inhibition of adipocyte markers (**c,d**).

Second, we focused on the activation of Ampk since a Smo-Ampk axis was recently identified to mediate Hh metabolic responses in adipose and muscular cells^14^. Of note, Ampk activation as well as its upstream kinase Lkb1 were both shown to reduce adipogenesis^54^,^55^,^56^. Indeed, we observed a robust phosphorylation of Ampk following 24 h treatment of 3T3-L1 preadipocytes with MP^Hh+^ and GSA-10, but not with SAG or recShh (Fig. 5a). To further characterize the involvement of Ampk signalling in adipogenesis inhibition by MP^Hh+^, we generated knockdown of key kinases implicated in Ampk signalling. Despite many trials, we were unable to produce an efficient knockdown of Ampk. We therefore turned towards upstream kinases Lkb1 and Camkk2 (calcium/calmodulin-dependent protein kinase kinase 2), which have Ampk as the main substrate and are both involved in the non-canonical Smo/Ampk axis identified in adipocytes^14^. Efficient knockdown (Kd) of Lkb1 abrogated MP^Hh+^ and GSA-10 effects on adipocyte differentiation (Fig. 5b). In contrast, MP^Hh+^ and GSA-10 maintained their inhibitory effects on adipogenesis in Camkk2 Kd cells (Fig. 5b). Accordingly, *PPAR*γ and *aP2* mRNA expression were restored in MP^Hh+^ and GSA-10-treated Lkb1 Kd cells (Fig. 5c), whereas they were still decreased in Camkk2 Kd adipocytes (Fig. 5d). It is noteworthy that the anti-adipogenic effects of SAG and recShh in minimal induction conditions were independent of Lkb1 or Camkk2, as illustrated by the conserved inhibition of PPARγ and aP2 mRNA expression in Lkb1 and Camkk2 Kd cells (Fig. 5c,d). Altogether, these results demonstrate that the anti-adipogenic effects of MP^Hh+^ or GSA-10 in 3T3-L1 preadipocytes is mediated *via* the activation of a Smo-Lkb1-Ampk pathway, previously evidenced in mature 3T3-L1 adipocytes^14^.

## Discussion

**Figure 6:**
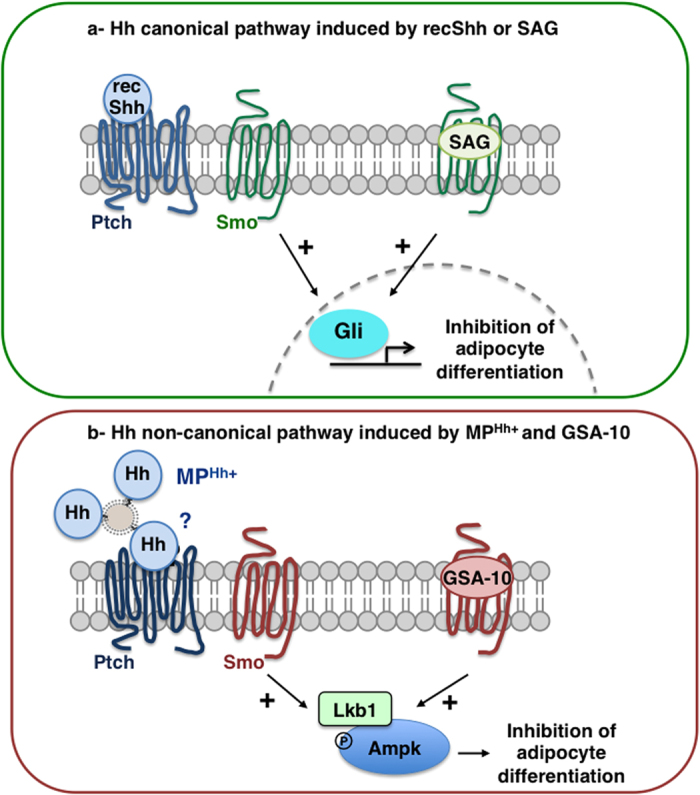
Proposed models of Hh signalling pathways used by recShh and SAG or MP^Hh+^ and GSA-10, respectively, to inhibit adipocyte differentiation of 3T3-L1 cells. (**a**) Hh canonical pathway induced by recShh and SAG. Non-lipidated form of Hh (recShh) or SAG by binding to Ptch and Smo, respectively, inhibits 3T3-L1 adipogenesis in minimal induction medium through Smo activation leading to Gli factors transactivation, and adipocyte differentiation inhibition.(**b**) Hh non-canonical pathway induced by MP^Hh+^ and GSA-10. MP^Hh+^ (by binding to Ptch ?) trigger a potent inhibition of adipocyte conversion similar to the inhibitory effects of recShh or SAG, but molecular mechanisms differ and involve non-canonical Hh signalling. Smo agonist GSA-10 recapitulates the hallmarks of MP^Hh+^-induced anti-adipogenic effects. Despite a Smo-dependency, MP^Hh+^ and GSA-10 effects are independent of increased transcription of Gli1 factors. Conversely, MP^Hh+^ and GSA-10 both rely on a Smo/Lkb1/Ampk axis to exert their anti-adipogenic effects, illustrating non-canonical Hh responses.

Adipogenesis, contributing to fat development, is one of the numerous developmental processes controlled by Hh signalling. In this work, we investigated the effect of MP-associated Hh (MP^Hh+^) on adipogenesis by studying their effects on the well-described 3T3-L1 adipocyte differentiation model. We show that MP^Hh+^ trigger potent inhibition of adipocyte conversion similar to the inhibitory effects of non-lipidated form of Hh using recShh or the Smo agonists SAG and GSA-10. However, we also demonstrate that MP^Hh+^ and GSA-10 specifically use a non-canonical pathway involving a Smo/Lkb1/Ampk axis to exert their anti-adipogenic effects, unlike recShh or SAG whose effects rely on Gli-dependent transcriptional activity (Fig. 6).

Different secreted forms of Hh morphogen are described in mammalian systems in particular, the EV-associated Hh forms, including exosomes and MP^18^,^22^,^23^,^24^,^25^,^26^,^57^. While MP refer to 150–1000 nm vesicles secreted following budding from the plasma membrane, exosomes indicate smaller vesicles (30–100 nm) released as a consequence of the fusion of multivesicular bodies with the cell surface. In our cellular model, we selectively target Hh to MP fraction secreted by T-CEM lymphocytes following apoptotic/activated stimuli, but not exosomes. Other authors also reported specific association of Hh on MP in hepatic stellate cells following specific activation signals^25^, suggesting that they can participate in pathophysiological responses to cell damages. No information is yet available regarding the Hh forms associated to MP. Since MP-associated Hh is derived from cellular plasma membranes, this suggests the presence of doubly-lipid modified Hh forms on MP. By analogy, lipoproteins released by flies or mammalian cell types contain lipid-modified forms of the morphogen^21^. Lipid modifications are required for cellular reception and influence both cellular concentration of Hh ligands and signalling potency^18^,^58^. However, recent work demonstrated that endogenous-derived Hh morphogens are modified by a heterogeneous subset of saturated and unsaturated fatty acids, which would influence their subcellular trafficking and activity^59^. Since MP are thought to be derived from lipid-enriched plasma membrane microdomains, the so called lipid-rafts^60^,^61^, which are highly enriched in saturated fatty acids, such membranous origin might influence fatty-acid speciation of Hh forms associated to MP.

In our model, in contrast to recShh, preincubation with 5E1 Ab did not antagonize neither the anti-adipogenic effects of MP^Hh+^ nor MP^Hh+^ internalization by preadipocytes. However, we can not exclude the possibility that these MP^Hh+^ could bind to Ptch at a site, which is not fully antagonized by 5E1 Ab. Knockdown of Ptch receptor in 3T3-L1 completely blocked adipogenic process and is associated with a constitutive activation of Gli factors (A.F., S.L.L. unpublished data), thus making this cell model unemployable for studying MP^Hh+^ effects on adipocyte differentiation in the absence of the receptor. Further investigations would therefore be needed to clearly define how MP^Hh+^ interact and transduce Hh signalling responses in recipient cells.

Hh signalling also depends on the combinatorial involvement of several co-receptors in addition to its cognate primary Ptch receptor^6^. Presence of co-receptors like proteoglycans and Lrp2 on a specific pool of exosomes was proposed to modify Hh signalling outcomes^26^. Similarly, accessory proteins like integrins, often retrieved on extracellular vesicles, might be necessary for the full signalling capability of Hh^26^. We cannot exclude that others proteins, in addition to Hh morphogen, may be specifically targeted within MP^Hh+^. Finally, internalized MP^Hh+^ could also regulate Hh signalling pathway in a manner mirroring lipoproteins-derived Smo-inhibitory lipids. Indeed, *Drosophila* studies reveal that Ptch destabilizes Smo at the membrane using lipids, recently identified as endocannabinoids^62^, derived from internalized lipoprotein particles^63^. The presence of Hh on lipoproteins would inhibit utilization of their lipids by Ptch, blocking the repressive function of lipoproteins-associated forms of Hh^63^.

By using antagonists that bind at different sites of Smo, we showed that, unlike SAG or recShh, MP^Hh+^ inhibitory effects were not reversed by most of Smo site 1-binding antagonists (cyclopamine or GDC-0449), nor site 2-binding antagonist (SANT-1) or combined site-1/site-2 antagonist (MRT-92). Similar antagonism profile was observed when Hh pathway was activated by GSA-10. Of interest, the anti-adipogenic effects of both MP^Hh+^ and GSA-10 were completely antagonized by LDE225, which was proposed to bind at the level of site 1^49^. Such results suggest that MP-associated Hh may correspond to the natural ligand that activates a Smo form identical to the one activated by GSA-10, but not by recShh or SAG. We previously showed that two activators of adenylate cyclase, forskolin and cholera toxin, potentiate GSA-10 differentiation effects on C3H10T1/2 cells^16^. In 3T3-L1 cells, we observed that GSA-10, like MP^Hh+^, retained its inhibitory effects even in the presence of IBMX, a phosphodiesterase inhibitor that maintains high intracellular levels of cAMP. One could therefore question the ability of cAMP to stabilize or potentiate such a Smo conformation. Finally, most of the antagonists tested in this study, like MRT-92, inhibit Smo trafficking in the cilium^37^, whereas they do not antagonize MP^Hh+^ anti-adipogenic effects. This suggests that MP^Hh+^ would activate a Smo conformation, which would mediate Hh downstream pathways independently of cilium translocation in agreement with the behaviour of a Smo conformation sensitive to GSA-10 identified in C3H10T1/2 cells^16^.

Our results provide evidence that natural Hh ligand carried by MP can activate a Smo/Lkb1/Ampk axis, disconnected from the canonical Hh signalling pathway, which will play a central role in metabolic responses. Lkb1 and Ampk proteins were shown to be concentrated at the basal-body of the cilium in kidney epithelial cells^64^ and identified as a complex with acetylated α-tubulin in co-immunoprecipitation experiments in 3T3-L1 mature adipocytes^14^. However, MP^Hh+^-induced Smo/Lkb1/Ampk axis displays some substantive differences with the pathway previously described in mature adipocytes^14^. First, SAG or recShh do not induce Ampk phosphorylation in preadipocytes and their anti-adipogenic effects are also not reversed by Lkb1 silencing. This is illustrated by the continuous decrease of PPARγ and aP2 mRNA expression in Lkb1 KD 3T3-L1 cells. Second, MP^Hh+^ or GSA-10 effects are independent of calcium flux modulation since silencing of Camkk2 in 3T3-L1 does not prevent their inhibitory effects. Third, we did not observe any change in acidification of culture media or in lactate concentrations following 24 h treatment with Hh signalling inducers on preadipocytes (A.F., S.L.L. unpublished data). These differential responses between preadipocytes and adipocytes might be related to the regulation of distinct metabolic pathways between the two stages of maturation and to the presence of distinct Smo conformations. Moreover, absence of the cilium in mature adipocytes may condition Hh signalling responses in favour of a non-canonical pathway not linked to this primary organelle.

Different publications already reported that activation of Ampk leads to inhibition of adipogenesis. In particular, 5-Aminoimidazole-4-carboxamide-1-β-d-ribofuranoside (AICAR), an activator of Ampk, was shown to inhibit fat accumulation in 3T3-L1 adipocytes by suppressing the expression of PPARγ and C/EBPα^55^,^66^. Such inhibition has been moreover related to the modulation of the Wnt/β-catenin pathway^56^. Furthermore, Lkb1/Ampk signaling was recently shown to inhibit adipocyte differentiation through its negative action on CREB-regulated transcription co-activators (CRTCs) and on Class IIa histone deacetylases, both being important regulators of PPARγ and C/EBPα expression^54^. Finally, recent work demonstrates that activation of Ampk reduces Gli1 protein stability and transcription activity in medulloblastoma, therefore compromising Hh canonical responses^65^. Our data showed that MP^Hh+^ anti-adipogenic effects are upstream of PPARγ rendering these different molecular mechanisms plausible to mediate MP^Hh+^-inhibitory effects.

The release of extracellular vesicles-associated Hh in *Drosophila*^18^,^24^,^67^ or in different mammalian cell types^22^,^23^,^25^ raises the question of their pathophysiological relevance *in vivo*. Circulating MP from healthy patients harbour Hh proteins^23^ however, to date, no report has studied the proportion of circulating Hh transported in association with extracellular vesicles. Yet, MP^Hh+^ release can be stimulated following specific signals suggesting their potential involvement in pathophysiological responses. We previously reported that systemic injection of MP^Hh+^ corrects angiotensin-II-induced hypertension and endothelial dysfunction in mice^68^, and exerts cardioprotection against ischemia reperfusion injuries in pigs^31^. Our current study identifies anti-adipogenic properties of MP^Hh+^. Considering the ability of Hh signalling to modulate fat metabolism through regulation of fat storage^34^,^35^, glucose uptake^14^ or mobilization of fat body lipids in *Drosophila*^69^, this raises the question of the ability of circulating MP^Hh+^ to control adipose tissue development *in vivo*. In addition, Smo-dependent non-canonical Hh signalling was reported to elicit various cellular responses ranging from Ca^2+^ signalling and cytoskeletal rearrangements to metabolic rewiring pathways^70^. The contribution of these non-canonical Hh signalling axes in comparison to Hh canonical pathways, in overall metabolism is still an open question. In this context, MP^Hh+^ as natural Hh non-canonical pathway inducers represent interesting biological tools to investigate the complexity of Hh responses events either linked to canonical or non-canonical Hh signalling.

In conclusion, despite complete adipogenesis blockade following recShh/SAG or MP^Hh+^/GSA-10 treatment of 3T3-L1 preadipocytes, molecular mechanisms behind these inhibitory effects are different and use canonical or non-canonical Hh signalling pathways, respectively (Fig. 6). This report suggests that MP^Hh+^ induce a signalling pathway involving Smo receptor in preadipocytes, which is independent of the increased transcription of Gli factors, and shares many pharmacological features with the Smo conformation activated by GSA-10, thus illustrating non-canonical Hh responses. This work therefore highlights novel therapeutic strategies based on MP^Hh+^ or GSA-10 agonist to modulate adipose tissue development and more largely the non-canonical Hh pathways at the whole organism level.

## Additional Information

**How to cite this article**: Fleury, A. *et al*. Hedgehog associated to microparticles inhibits adipocyte differentiation *via* a non-canonical pathway. *Sci. Rep*. **6**, 23479; doi: 10.1038/srep23479 (2016).

## Supplementary Material

Supplementary Information
