# Supplementary material for: Hedgehog associated to microparticles inhibits adipocyte differentiation via a non-canonical pathway
Source: Sci Rep. 2016 Mar 24;6:23479. doi: 10.1038/srep23479 (PMC4806302; doi:10.1038/srep23479)
Supplement: Supplementary Information [file srep23479-s1.pdf]

Supplementary information

Hedgehog associated to microparticles inhibits adipocyte differentiation *via* a non-canonical pathway

Audrey Fleury, Lucile Hoch, M. Carmen Martinez, Hélène Faure, Maurizio Taddei, Elena Petricci, Fabrizio Manetti, Nicolas Girard, Andre Mann, Caroline Jacques, Jérôme Larghero, Martial Ruat, Ramaroson Andriantsitohaina and Soazig Le Lay

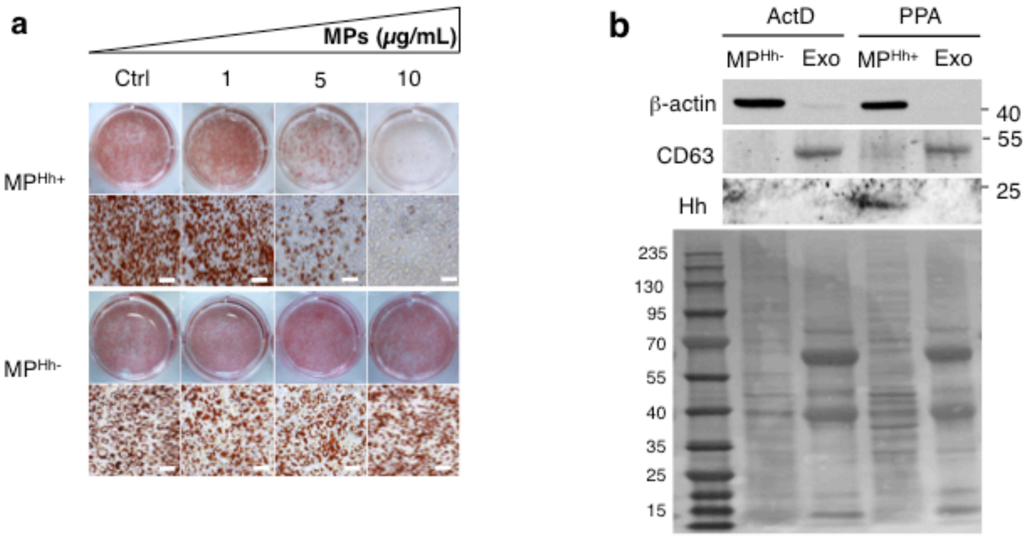

Figure S1. *MP^{Hh+}, and not exosomes, inhibit adipocyte differentiation of 3T3-L1 in a dose-dependent manner.*

a) 3T3-L1 cells were incubated with indicated concentrations of MP^{Hh+} or MP^{Hh-}, in classical induction medium, with a medium change every 24h. Cells were stained with ORO on day 6-8 of differentiation. Representative ORO-stained images are shown. Scale bar: 50 µm.

b) Hh morphogen specifically associates with the MP fraction and not with the exosomal fraction secreted by activated/apoptotic CEM T lymphocytes. MP and exosomal fractions (Exo) were respectively isolated from ActD or PMA/PHA/ActD (stated as PPA)-stimulated CEM T lymphocytes supernatants by using different centrifugations steps. MP^{Hh-} correspond to MP isolated from ActD-stimulated CEM T cells, whereas MP^{Hh+} are the MP isolated from PPA-stimulated CEM T lymphocytes. Immunoblot shows the unique presence of Hh in MP^{Hh+}. Actin and CD63 are shown as respective protein markers of MP and exosomal fractions, respectively. Ponceau red staining of the membrane is presented to illustrate sample loading.

c) Exosomes derived from activated/apoptotic CEM T lymphocytes do not alter adipocyte differentiation, in agreement with the absence of Hh. Lipid accumulation was visualized by ORO staining on day 6-8 of differentiation after exposing 3T3-L1 cells (every 24h) to 10µg/mL exosomes in classical or minimal induction media. None of exosomal preparations, either derived from ActD or PMA/PHA/ActD (stated as PPA)-stimulated CEM T lymphocytes supernatants - which are both devoid of Hh (see panel b)- alter 3T3-L1 adipocyte differentiation process. Scale bar: 50 µm.

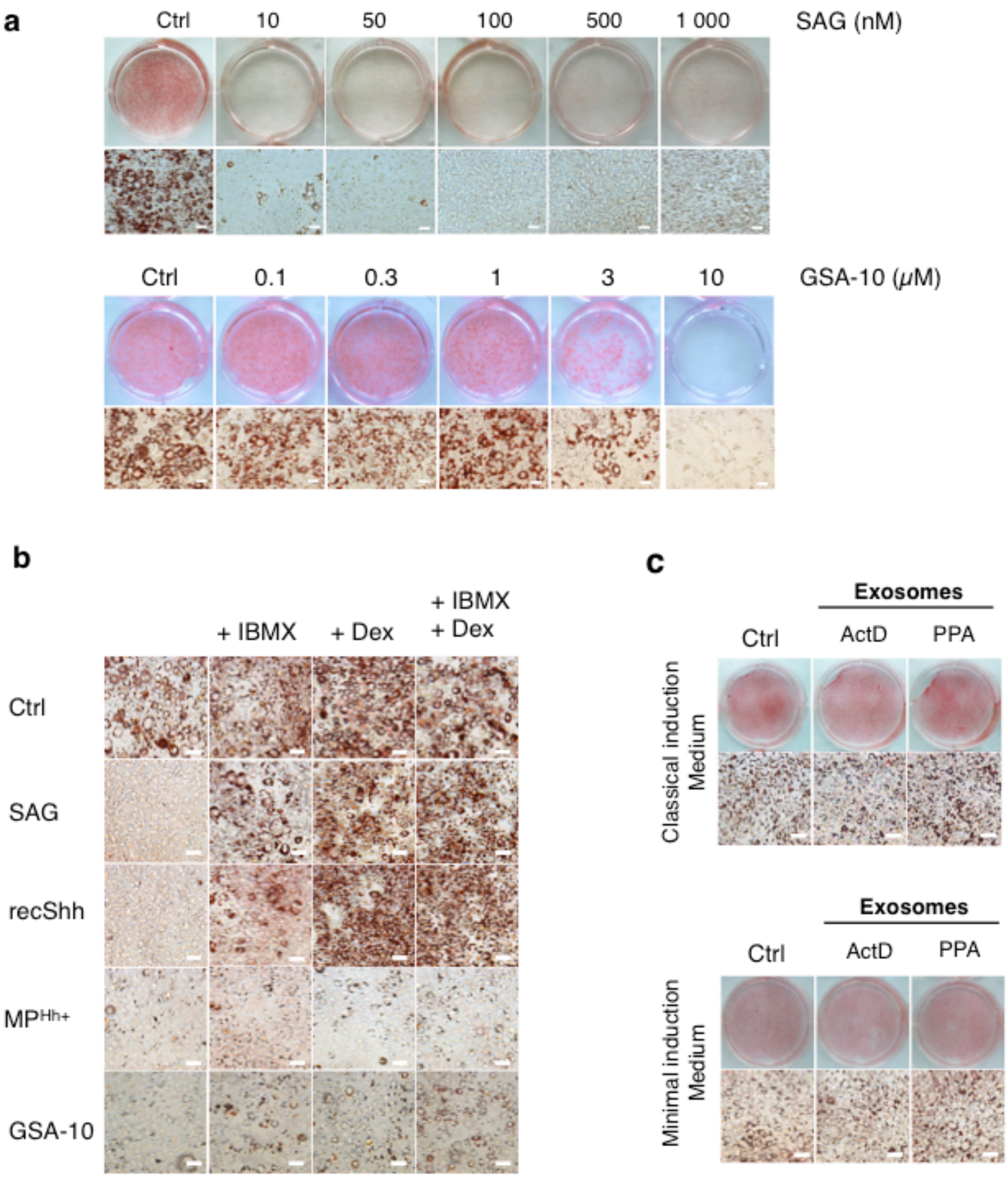

Figure S2. *Differential inhibition of adipocyte differentiation by Smo agonists, depending on culture medium conditions.*

a) Dose-response of SAG and GSA-10 on 3T3-L1 adipocyte differentiation in minimal induction medium. Indicated concentrations of SAG (upper panel) or GSA-10 (lower panel) were applied to 3T3-L1 preadipocytes for 72h in minimal induction medium, and cells were stained with ORO on day 6 of differentiation. Representative ORO-stained images are shown. Scale bar: 50µm.

b) Addition of Dex or IBMX to minimal induction medium is sufficient to abolish the inhibitory effects of SAG and recShh on adipogenesis as exemplified by ORO staining. Representative ORO images are shown. Scale bar: 50 µm.

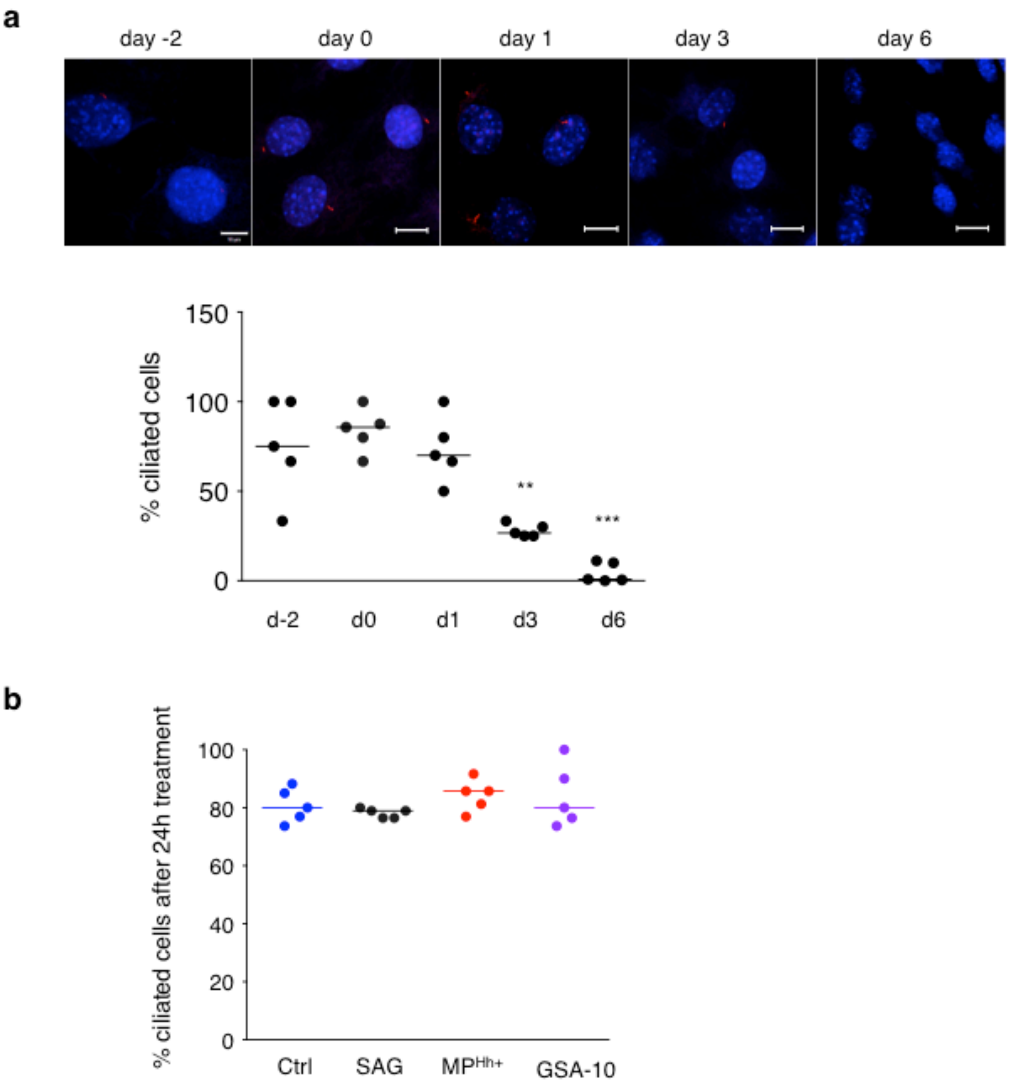

Figure S3. *MP^{Hh+} do not affect cilium presence in 3T3-L1 cells*

a) Representative confocal images during 3T3-L1 differentiation course (from day-2 to day 6) illustrating the transient presence of a primary cilium (anti-acetylated tubulin, red) only in preadipocyte stages. Nuclei of cells are stained with DAPI (blue). Percentage of ciliated cells was calculated based on the ratio of acetylated-tubulin staining to the total cells evaluated by DAPI staining, *n*=3-5 independent microscopic fields observed. Note the total absence of cilium in differentiated adipocytes (day 6). Scale bar: 10 µm.

b) Treatment of 3T3-L1 cells with Hh signalling activators does not alter cilium presence. Presence of primary cilium per cell after 24h treatment of post-confluent 3T3-L1 (day 0) with SAG (200 nM), MP^{Hh+} (10 µg/mL) or GSA-10 (10 µM) was observed. Data are presented in % of ciliated cells after 24h, *n* = 3 independent microscopic fields observed.

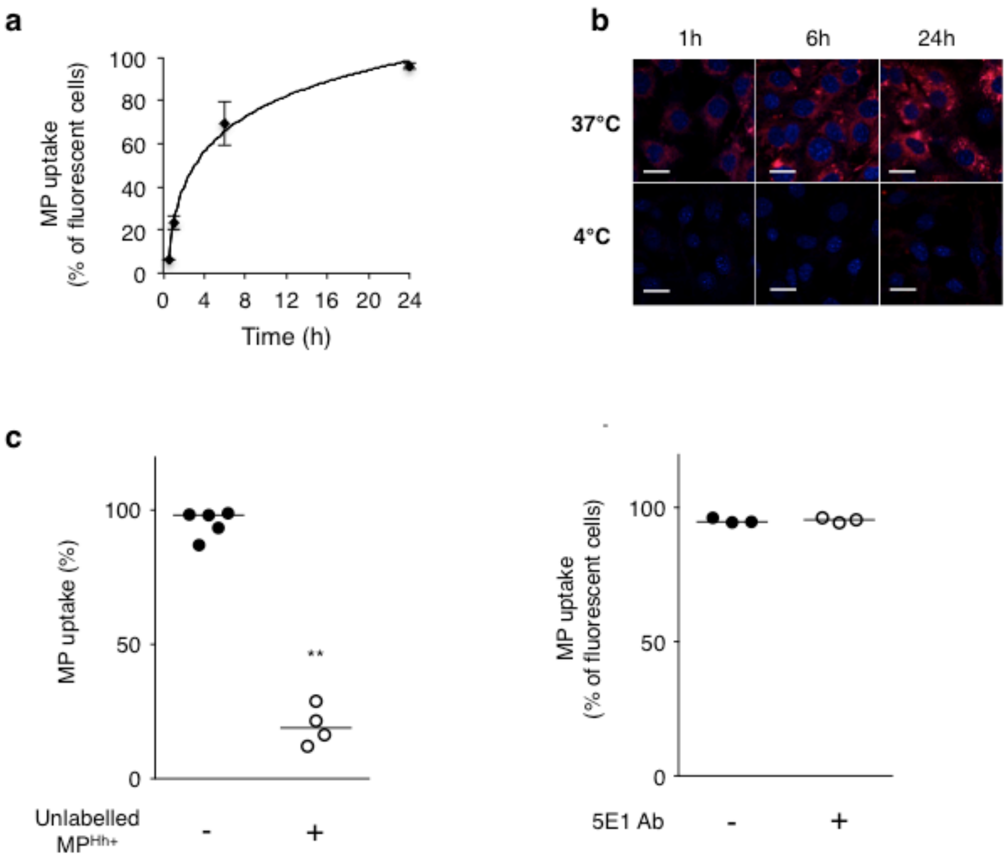

Figure S4. *MP^{Hh+} internalization is not blocked by preincubation with 5E1 antibody*

a) Time-dependent uptake of PKH26-labeled MP^{Hh+} investigated by flow cytometry. 3T3-L1 cells incubated with 10 µg/mL PKH26-labeled MP^{Hh+} were analysed at different time points (1h, 2h, 6h and 24h). Percent of fluorescent 3T3-L1 cells were determined by flow cytometry by detection of PKH26-labeled MP^{Hh+} uptake in cells.

b) Time-dependent confocal microscopy of PKH26-labeled MP^{Hh+} uptake at 37°C or 4°C in 3T3-L1 cells. Confocal images of PKH26-labeled MP^{Hh+} (10 µg/mL) accumulation over time (1h, 6h and 24h) at 37°C. This is totally abolished when cells are placed at 4°C. Representative images are shown, *n*=2 independent experiments (scale bar: 10 µm).

c) PKH26-labeled MP^{Hh+} uptake in the absence or presence of excess unlabelled MP^{Hh+}. Competitive uptake of 10 µg/mL PKH26-labeled MP^{Hh+} was assessed by adding a five-fold excess of unlabelled MP^{Hh+}. Uptake of MP after 1h was quantified by flow cytometry. Fold regulation is relative to cells treated with 10 µg/mL PKH26-labeled MP^{Hh+} alone.

d) Flow cytometry analysis of PKH26-labeled MP^{Hh+} uptake preincubated with or without 5E1 antibody. PKH26-labeled MP^{Hh+} preincubated with or without 10 µg/mL 5E1 Ab for 30min, were incubated for 24h on 3T3-L1 cells. Fold regulation in MP uptake is relative to cells treated with 10 µg/mL PKH26-labeled MP^{Hh+} alone. All results are presented as scatterplots plus median, *$p<0.05$.

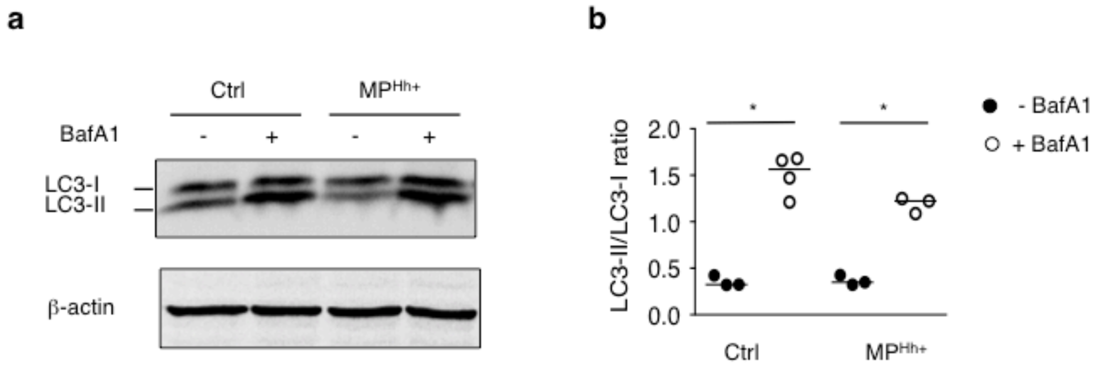

Figure S5. *MP^{Hh+} do not modulate autophagic flux*

a-b) LC3 immunoblot (a) of 3T3-L1 lysates treated with 10µg/mL MP^{Hh+} in the presence or absence of 100nM bafilomycin A1 (BafA1) for 2h. Autophagic flux quantification (b) was based on measurement of LC3-II/LC3-I ratio in the absence or presence of BafA1. β-actin was used as loading control. No change in autophagic flux was observed following treatment with MP^{Hh+}. All results are presented as scatterplots plus median, *$p<0.05$.

Figure 1. panel a

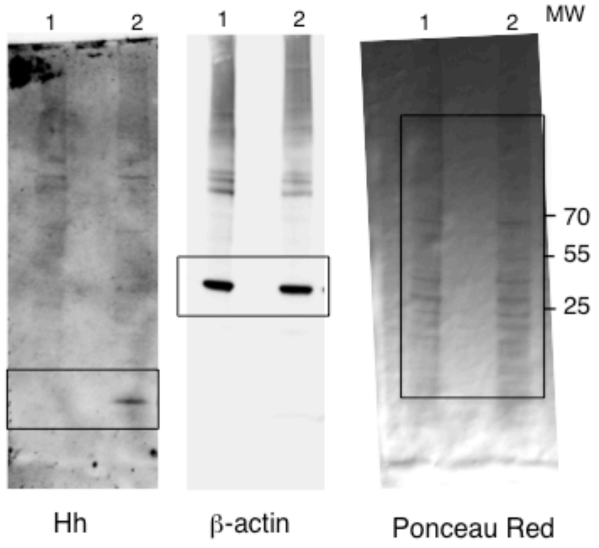

1- MP^{Hh-}

2- MP^{Hh+}

MW Molecular weight (kDa)

Figure 1. panel h

1- Ctrl

2- MP^{Hh+}

3- MP^{Hh-}

MW Molecular weight (kDa)

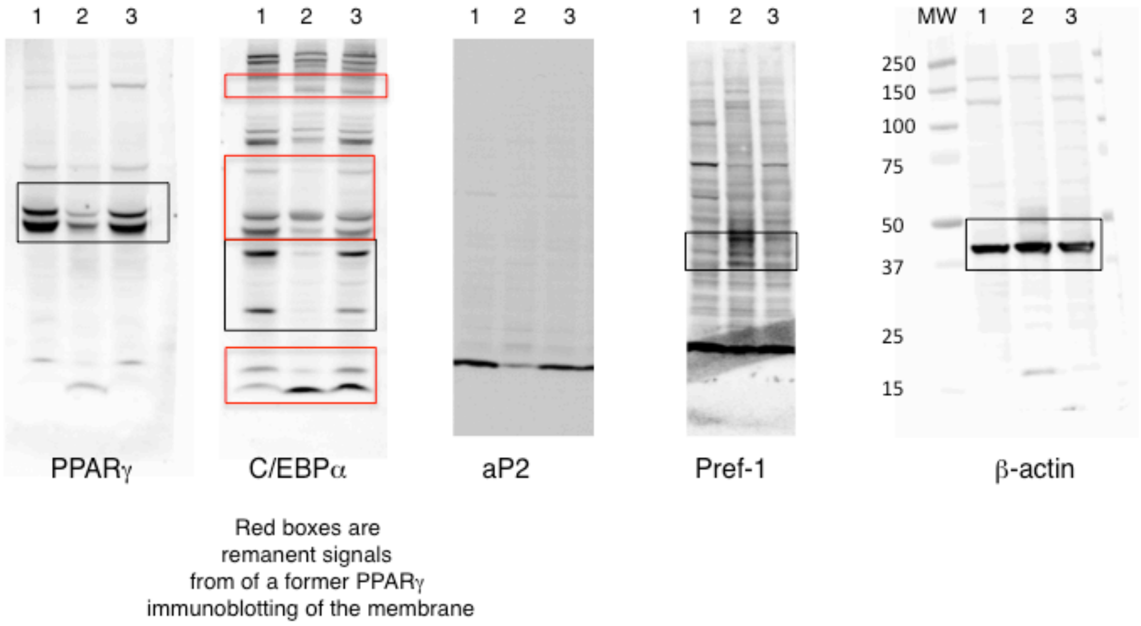

Figure S6. *Unprocessed images of the key immunoblots of Figure 1*

Boxes indicate image areas shown in the indicated panels.

Figure 2. panel b

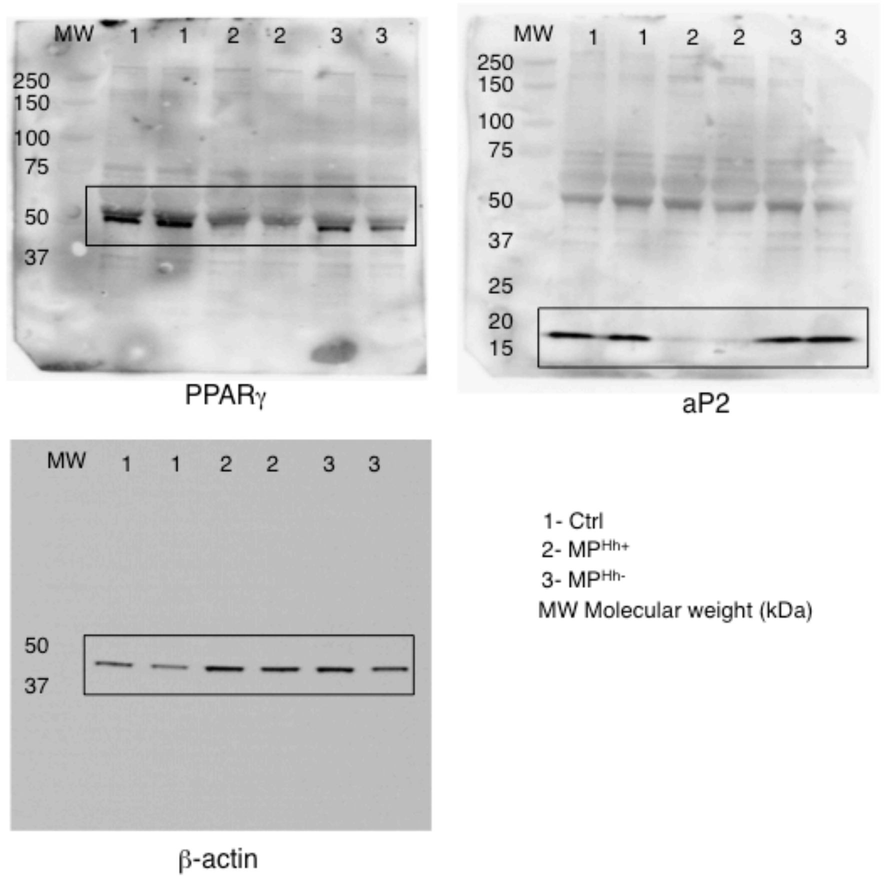

Figure S7. *Unprocessed images of the key immunoblots of Figure 2*

Boxes indicate image areas shown in Figure 2b.

Figure 5. panel a

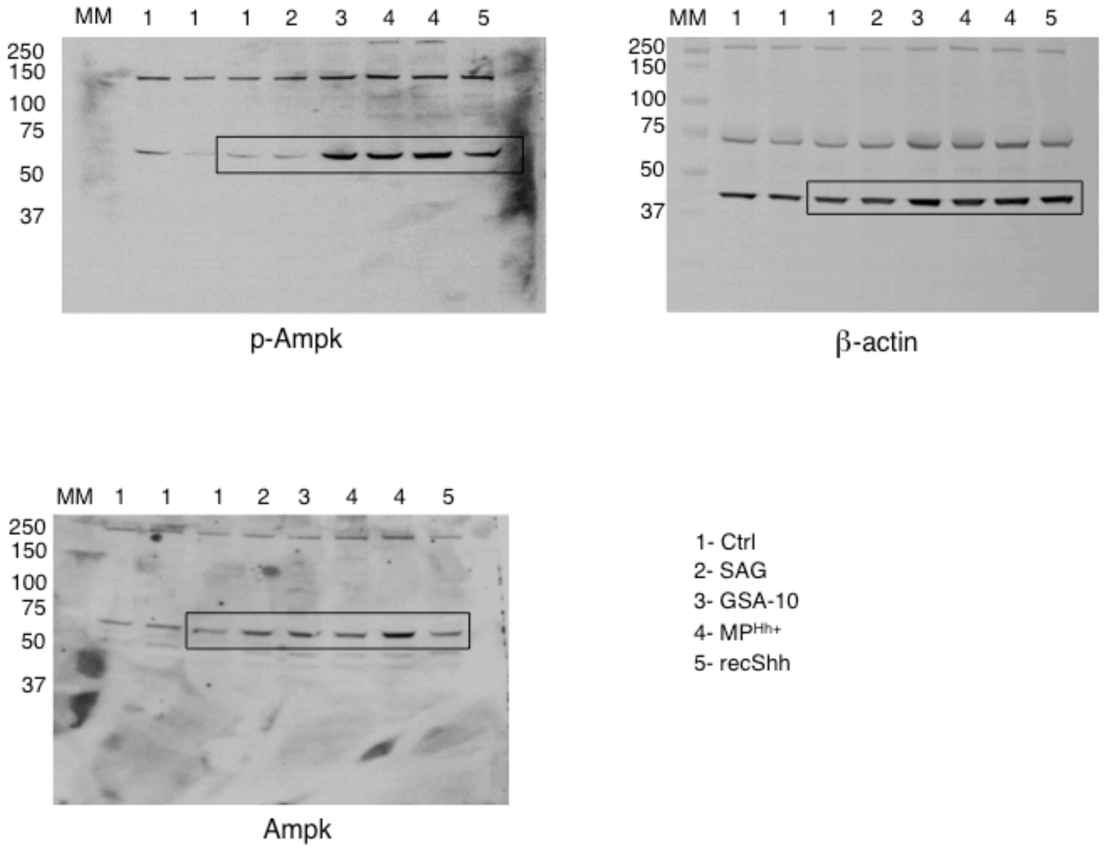

Figure S8. *Unprocessed images of the key immunoblots of Figure 5*

Boxes indicate image areas shown in Figure 5a.

**Primers used**	**Forward (5'-3')**	**Reverse (5'-3')**
36B4	TCCAGGCTTTGGGCATCAC	CTTTATCAGCTGCACATCAC
Ampkα1	ACGGCCGAGAAGCAGAAGCAC	TCGTGCTTGCCCACCTTCAC
aP2	AACACCGAGATTTCCTT	ACACATTCCACCACCAG
Camkk2	CCGTTCGAAGGTAGCCGCCG	CGCTGCCTTGCTTCCTTGGGC
CEBPα	TCTGCGAGCACGAGACGTC	GCCAGGAACTCGTCGTTGAA
CEPBβ	GCAAGAGCCGCGACAAG	GGCTCGGGCAGCTGCTT
CoupTFII	TCATCTCCTCTGCCAAGCATC	TCTGAGTGAGGCACACGAAGC
GATA2	TGCAACACACCACCCGATACC	CAATTTGCACAACAGGTGCCC
Gli1	CAGGGTCCCAGGGTTATGG	AGGTCGAGGCTGGCATCAG
Lkb1	TGACTGCGGCCCACCACTCT	CGGAACCAGCTGTGCTGCCTA
Ptch1	TGACGGGGTCCTCGCTTACAA	CTACCAGACGCTGTTTAGTCA
PPARγ	AGGCCGAGAAGGAGAAGCTGTTG	TGGCCACCTCTTTGCTCTGCTC
Pref-1	CGAAATAGACGTTCGGGCTT	TCGTACTGGCCTTTCTCCAG
Runx2	ACGTCCCCGTCCATCCA	TGGCAGTGTCATCATCTGAAATG
Smo	GAGCGTAGCTTCCGGGACTA	CTGGGCCGATTCTTGATCTCA

Table S1. Primers used for quantitative real Time-PCR.
